# Prevalence of Prediabetes, Diabetes and Its Predictors among Females in Alkharj, Saudi Arabia: A Cross-Sectional Study

**DOI:** 10.5334/aogh.2467

**Published:** 2019-07-22

**Authors:** Jamaan M. Al-Zahrani, Abdulrahman Aldiab, Khaled K. Aldossari, Sameer Al-Ghamdi, Mohammed Ali Batais, Sundas Javad, Shanila Nooruddin, Nida Zahid, Hira Abdul Razzak, Ashraf El-Metwally

**Affiliations:** 1 Family Medicine Department, College of Medicine, Prince Sattam Bin Abdulaziz University, Al-Kharj, SA; 2 Internal Medicine Department, College of Medicine, King Saud University, Riyadh, SA; 3 College of Medicine, King Saud University, Riyadh, SA; 4 College of Public Health and Health Informatics, King Saud Bin Abdulaziz University for Health Sciences, Riyadh, SA; 5 Dow University of Health Sciences, Karachi, PK; 6 Aga Khan University Hospital, Karachi, PK; 7 Ministry of Health and Prevention, Dubai, AE

## Abstract

**Background/Objective:**

The prevalence of prediabetes and diabetes is reaching epidemic proportions across the globe. Therefore, this study aims to determine the prevalence of prediabetes and diabetes, together with its accompanying risk factors, among young females.

**Methods:**

An exploratory cross-sectional survey was conducted with 638 Saudi females in Alkharj, Saudi Arabia. Statistical analysis was carried out using STATA version 14. Odds ratios for the risk of diabetes and associated factors were calculated using log-binomial and multinomial logistic regression. Standardized prevalence and strata-specific prevalence of diabetes and prediabetes for different risk factors were also calculated.

**Findings:**

The study revealed that nondiabetics and prediabetics were more prevalent between the ages of 18 and 24 years, while diabetic patients were consistently between 25 to 44 years of age. The average value for HbA1c in females was 5.44. Regression analysis shows that being older, married, obese, a smoker or less educated significantly increases the risk for both prediabetes and diabetes. Mutivariable analysis revealed obesity had a significant association with both prediabetes and diabetes. Prediabetics were 2.35 times more likely to be obese as compared to nondiabetics, with 95% CI (1.38–3.99). Similarly, diabetics were 6.67 times more likely to be obese compared to nondiabetics 95% CI (1.68–26.42).

**Conclusion:**

Our study shows the prevalence of diabetes and prediabetes among females from Al Kharj was 3.8% and 18.8%, respectively. The diabetic and prediabetic female participants had higher mean BMI and waist circumference, were older in age, were married, and smoked as compared to nondiabetics. In the context of the findings of our study, and keeping in view the the burden of this disease globally and in our population, it has now become extremely important to understand these factors and encourage health-promoting behaviors to construct effective interventions.

## Strengths and limitations of this study

- The prevalence of diabetes is important in public health to assess the burden of disease in a specified population, as well as in planning and allocating health resources.
- The diagnosis of DM was performed using HbA1c, which has been used as an authentic marker for evaluating the presence of diabetes.
- The collected data was cross-sectional; causal relationships cannot be established, and the observed association may be because of other unexplored factors.

## 1. Introduction

Diabetes mellitus (DM) is a progressive and chronic illness that affects all age groups and has become a serious, global public healthcare concern [1]. The International Diabetes Federation (2016) stated that 415 million individuals are suffering from this condition, and it is predicted that it will reach 642 million by 2040 [2]. Diabetes is a “metabolic condition,” which is characterized by “high blood glucose/sugar levels” that result in severe and worsening insulin resistance [3]. The most common types of diabates include Type 1 diabetes mellitus, Type 2 diabetes mellitus, and gestational diabetes. In addition, there are several other kinds of diabetes that present a diagnostic challenge for healthcare practitioners. LADA is an auto-immune diabetes having a later onset age and slower progression toward dependence of insulin than is observed in most of the Type 1 diabetes patients. As they do not require insulin initially at diagnosis, LADA patients may be misdiagnosed frequently as having Type 2 diabetes mellitus [4].

The American Diabetes Association in 2015 formerly equated prediabetes with the World Health Organizations’s (WHO) intermediate hyperglycemia; however, currently borderline HbA1c levels are also an important indicator of prediabetes [5]. Yet, specific mechanisms leading to diabetes are not known [6]. In 2016, a study conducted in Florida revealed that 7.3% and 12.1% of adults in the study were diagnosed with prediabetes and diabetes, respectively [7].

Prediabetes is also referred to as intermediate hyperglycemia or borderline diabetes. Screening analysis comprises, but is not limited to, “casual plasma glucose level,” “75-g oral glucose tolerance test” (OGTT), and “fasting plasma glucose (FPG) [8,9].” Its worldwide prevalence is rising, and it is estimated that by 2030 more than 470 million individuals will be prediabetic [10]. The condition occurs when pancreatic beta cells are incapable of producing an adequate amount of insulin for disposing of blood glucose, or an individual’s body is incapable of using insulin well enough for reducing the blood glucose levels, or because of pancreatic beta cells failing to discharge enough insulin.

The genetic architecture of quantitative indices for insulin resistance and beta cell functions varies greatly among both nondiabetics and diabetics [11]. This indicates that beta cell function often tends to supersede insulin resistance as a crucial factor of Type 2 diabetes [12]. Additionally, obesity is a critical insulin resistance determinant, where adiposity modulates the genetic aspects of insulin resistance, thus contributing to the Type 2 diabetes heterogeneity [13]. Hence, adiposity levels often have a potential to perturb the psychological milieu where genetic variants in insulin signalling pathways operates. Hence, fat in the pancreas, genetic variants, and major glucose recipient organs exacerbate insulin resistance that consequently impairs the beta cell functions. Obesity also increases the demands for insulin, due to which beta cell hyperfunction may result in exhausting beta cells, resulting in their dysfunction [14].

The risk for Type 2 DM increases due to prediabetes, which results in the susceptibility of different diabetes-related complications, such as eye problems, kidney failure, nerve damage, stroke, and heart disease [15]. However, literature indicates that lifestyle modification for prediabetic patients can help reduce or prevent diabetes progression by 40–70%. This finding highlights the necessity for early diagnosis of diabetes [16,17]. A study conducted in Florida suggested in the United States approximately 86 million people over 20 years of age were prediabetic [7].

Middle Eastern countries have a specifically high prevalence of both the conditions for prediabetes and diabetes mellitus. According to the WHO country profile in 2016, the prevalence of diabetes in the Saudi population was 14.4% (females: 13.8%). Of this number, 33.7% were obese (females: 39.5%) and 68.2% were overweight (females 69.2%). It was determined that these conditions contributed to 5% of overall deaths in the same region [18]. DM is irrevocable if proven. It develops gradually but progresses consistently and might take years to develop from a prediabetic to a diabetic state, regardless of any interventions. With increasing demographic and epidemiologic transitions impacting the individual, rapid and accelerating urbanization, and subsequent modifications in physical activity, diet, and lifestyle [19], it is imperative to monitor the unremitting diabetes burden among young females in Saudi Arabia. This may be achieved by using rigorous diagnostic procedures, as well as assessing risk factors with an aim to create efficient control and prevention strategies [20]. According to the Centers for Disease Control (CDC), social determinants of health are characterized as economic and are social conditions that principally influence individual health and communities. Socioeconomic factors influence health, such as mortality and outcomes in patients with diabetes [21].

Current epidemiological research studies in Saudi Arabia have determined such a state often perceives the effects of this emerging public health concern [22]. Previous evidence revealed abnormal glucose metabolism is recurrent among the Saudi population, with females showing prediabetes hallmarks [23]. A study conducted in Jeddah in 2015 demonstrated the prevalence of prediabetics was 9.0%: 9.4% in men and 8.6% in women. For DM, the results indicated the prevalence was 12.1%: 12.9% in men and 11.4% in women [1]. A separate study conducted among the Saudi military community concluded that the average BMI was 27.6 kg/m^2^. Almost two thirds of the population in the study were either “overweight (34.7%)” or “obese (29.9%).” The mean waist circumference was 94.7 cm [24].

Being a high-income state with a well-built health care infrastructure, adequate screening and diagnostic tools are available in the primary health care centers countrywide. Given the high frequency of individuals with diabetes, it is essential to determine the prevalence of prediabetics at a younger age so that the risk of DM can be minimized, which would lead to less economic burden on the health system and the individual [25]. The Diabetes Prevention Program demonstrated that around 11% of individuals with prediabetes developed Type 2 diabetes every year during the average 3 years of follow-up [26]. However, the conversion rate varies with prediabetes definition and population characteristics. In 2014, it was projected that the cost associated with diabetes was approximately 17 billion Riyals. If those who were not diagnosed as having DM were combined, the future treatment cost would escalate to 27 billion Riyals. Moreover, if those with prediabetes progress to become diabetics at the present conversion rate, then the future cost would be around 43 billion Riyals [27].

Regardless of the rising DM burden with respect to the Saudi population, current data on its epidemiology is limited. This type of evidence, however, is important for intervention planning. The present study, therefore, aims to investigate both the prevalence and the determinants of prediabetes and diabetes among the Al-Kharj female population so that strategies can be developed for early intervention for prediabetes and to prevent development of diabetes and its associated complications.

## 2. Methodology

### 2.1. Study Design

An exploratory cross-sectional study was performed.

### 2.2. Sample Selection

“Al Kharj is a city in Al Kharj Governorate in central Saudi Arabia located around 77 km south of Riyadh.” It is one of the main centers in the “Kingdom of Saudi Arabia” for dairy, as well as an agriculture industry supported with modern administration and economic significance. The region possesses natural resources, a geographical location of high importance, racial diversity, and strong population density. Furthermore, the residing population represents different racial groups. The population under examination for this study consisted of apparently healthy female Saudi adults at least 18 years of age during the time of recruitment.

### 2.3. Sample Size and Sampling Technique

The sample size was estimated on open Epi software version 3.01. The minimum sample size of 638 females was based on the anticipated prevalence of DM ranging from 18.6% to 56% [28], required precision of 5%, a level of significance of 5%, a design effect of 1.5, while also considering a 10% nonresponse rate. We have reported the female part of the study. Separate analysis was performed for males due to a difference in age structure as reported in the previous study [29].

Multi-stage sampling was used, carrying out participant selection from different government or private institutes. Two strata were constructed, classifying it into private and public institutes. A total of 25 institutes were identified in the region. These institutes were divided into two strata. We selected three institutes randomly, including one public university, one governmental organization, and one private institute through cluster sampling. Another cluster sampling was done on the selected institutes. Sampling units were then selected using simple random sampling from each of the institutes, using the list of respondents obtained from each department of the institutes.

### 2.4. Inclusion and Exclusion Criteria

The inclusion criteria included Saudi female citizens who were at least 18 years of age. However, those participants who did not give informed consent were excluded from the study.

### 2.5. Materials/Instruments

For the collection of data, two trained physicians interviewed respondents using a 20-item Arabic questionnaire. The questionnaire being used was grounded on a review of the evidence published in prior sources. The following information was collected: the first part included demographics (such as age, education, employment, and marital status), the second part included relevant medical data (smoking, chronic medical illness, chronic pain, and GHQ-12 scores), and the last part consisted of physical parameters (weight, height, waist circumference, blood pressure, and pulse). All participants completed a self-administered questionnaire, followed by a physical examination and blood test.

The GHQ-12 was used in other studies with diabetic subjects and is a valid and reliable instrument that mainly segregates the population of subjects into those with psychiatric/mental illness and those without, making the achievement of our aims and objectives possible [30,31,32].

### 2.6. Physical Measurement

Well-trained nurses gathered the anthropometric weight measurement, waist circumference, height, blood pressure, and pulse. Height was measured to the adjacent 0.5 cm without shoes. Waist circumference was measured to the nearest 0.5 cm using a measuring tape. Weight was measured to the nearest 100 g, without shoes and with the subject wearing lightweight clothes. A “Health O Meter Digital Scale” (made in the USA), which reads to the nearest 100 g, was used for weighing. A specific scale was utilized to weigh all respondents. The scale was calibrated regularly, and zero was guaranteed before the weight of any participant was taken. Blood pressure and pulse were calculated using an automated and calibrated machine and using measurement criteria according to the JNC 8 guideline [33].

### 2.7. Body Mass Index

Body mass index (BMI) was calculated as weight in kilograms divided by height in meters squared (kg/m^2^) for all participants being studied. Normal (BMI < 25), overweight (25 < BMI < 30) and obese (BMI > 30) groups were determined based on widely used cut-off values for assessing obesity according to WHO criteria [34].

### 2.8. Waist Circumference

Waist circumference (WC) was used as a surrogate for abdominal obesity, and a WC value less than 88 cm was considered normal in women [35].

### 2.9. Blood Test

Blood samples were collected from each participant by trained nurses or phlebotomists. The patient was given a unique ID (barcode). The samples were drawn and sent to the hospital laboratory for blood analysis. These routine laboratory test samples were processed immediately. A Beckman Coulter AU analyzer was used to examine HbA1c.

### 2.10. Prediabetes and DM Definition

Prediabetes was well-defined using HbA1c cutoff level of 5.7– < 6.5% and DM ≥ 6.5%, as per the American Diabetes Association 2016 [36]. Participants with self-reported diabetes were also included during analysis to estimate true prevalence in the population.

### 2.11. Data Analysis

The general and baseline characteristics between prediabetic, diabetic, and nondiabetic individuals were reported using mean (standard deviation) for continuous variables and frequency and percentages for categorical variables. Significance of differences was also assessed using χ^2^ test for categorical variables and t-test or ANOVA for continuous variables. Additionally, prevalence (%) of various risk factors in participants with diabetes and prediabetes was reported. We calculated odds ratios (OR) using log-binomial regression and multinomial logit regression for estimating diabetes risk and its association with different risk factors, such as anthropometric measures and lifestyle factors. A p-value of <0.05 was considered significant throughout the study. All statistical analysis was performed using statistical software StataCorp STATA version 12.

### 2.12. Ethical Approval

The study protocol was approved by the local institutional review board (i.e., Committee of Scientific Research and Publication). Written informed consent was attained from all the study participants aged 18 years and above. Furthermore, the identity of the respondents, together with their personal information, was kept confidential. It was ensured by the researcher that information attained by the participants will not be utilized in the future for any other purposes.

### 2.13. Patient and Public Involvement

This study was supported by an advisory group including representatives of employees and students who provided input into the design of research. This advisory board met on a regular basis to make sure that methods used were acceptable. At the end of the study, this advisory group commented on the findings and contributed to the dissemination plan.

## 3. Results

Table 1 presents baseline characteristics of the study participants by their diabetes status and for total population. A total of 638 participants were recruited in the study. Out of which, 24 (3.7%) were diabetics, 120 (18.8%) were prediabetics, and 494 (77.4%) were nondiabetics. The mean HbA1c was 5.44 (SD = 0.72, range = 2.3–12.7). About 619 participants had HbA1c levels below 6.5%, and 3 participants had HbA1c levels above 10%. The mean HbA1c levels were significantly higher among diabetic females (7.72, SD = 2.12) versus prediabetics (5.87, SD = 0.17) and nondiabetics (5.23, SD = 0.32) (p-value < 0.0001). The mean age of the study participants was 23.36 (SD = 6.5), with a range from 18 to 60 years. About 80% of the participants were between 18 and 24 years, 18% were between 25 and 44 years, and only 2% were between 45 and 60 years. Individuals with diabetes had a significant higher mean age (34.5, SD = 10.6) as compared to the prediabetes individuals (26.4, SD = 8.6) and nondiabetics (22.08, SD = 4.58) (p-value < 0.0001). Among prediabetics, about 61.7% were 18 to 24 years, while 33.3% of participants were 25 to 44 years. However, a higher proportion of diabetics (50%) were 25 to 44 years, while only 29.2% were 18 to 24 years. About 46.2% and 38.5% prediabetics and diabetics, respectively, were 45 to 60 years.

**Table 1:** General characteristics of participants by diabetic classification in a female Al Kharj study population.

	Total (n = 638)	Total Nondiabetes (n = 614)	Nondiabetes (n = 494)	Prediabetes (n = 120)	Diabetes (n = 24)	P-value For trend**

*HbA1c	5.44 *(0.72)*	5.35 (0.39)	5.23 (0.32)	5.87 (0.17)	7.72 (2.12)	<0.0001
*Age in years	23.36 *(6.47)*	22.93 (5.85)	22.08 (4.58)	26.39 (8.63)	34.46 (10.58)	<0.0001
**Age groups (years)**						<0.0001
- 18–24	79.78 *(509)*	81.76 (502)	86.64 (428)	61.67 (74)	29.17 (7)	
- 25–44	18.18 *(116)*	16.94 (104)	12.96 (64)	33.33 (40)	50.00 (12)	
- 45–60	2.04 *(13)*	1.30 (8)	0.40 (2)	5.00 (6)	20.83 (5)	
*Body Mass Index in kg/m^2^ (BMI)	25.68 *(6.25)*	25.43 (6.07)	24.77 (5.79)	28.14 (6.42)	32.09 (7.41)	<0.0001
**BMI categories**						<0.0001
- Normal	54.08 *(345)*	55.70 (342)	60.32 (298)	36.67 (44)	12.50 (3)	
- Overweight	23.35 *(149)*	23.62 (145)	23.08 (114)	25.83 (31)	16.67 (4)	
- Obese	22.57 *(144)*	20.68 (127)	16.60 (82)	37.50 (45)	70.83 (17)	
*Waist Circumference in cm (WC)	75.90 *(13.70)*	75.24 (13.09)	73.55 (12.40)	82.18 (13.57)	93.23 (18.06)	<0.0001
**Waist Circumference**						<0.0001
- ≤88 Cm	82.29 *(525)*	83.71 (514)	86.84 (429)	70.83 (85)	45.83 (11)	
- >88 Cm	17.71 *(113)*	16.29 (100)	13.16 (65)	29.17 (35)	54.17 (13)	
**Smoking Status**						0.311
- Never Smoker	99.06 *(632)*	99.19 (609)	98.99 (489)	100 (120)	95.83 (23)	
- Ex-smoker	0.16 *(1)*	0.16 (1)	0.20 (1)	0	0	
- Current Smoker	0.78 *(5)*	0.65 (4)	0.81 (4)	0	4.17 (1)	
**Marital Status**						<0.0001
- Married	21.63 *(138)*	19.87 (122)	16.60 (82)	33.33 (40)	66.67 (16)	
- Single	78.37 *(500)*	80.13 (492)	83.40 (412)	66.67 (80)	33.33 (8)	
**Education Level**						<0.0001
- Primary	1.72 *(11)*	1.30 (8)	0.40 (2)	5.00 (6)	12.50 (3)	
- Secondary	5.02 *(32)*	5.05 (31)	4.05 (20)	9.17 (11)	4.17 (1)	
- Intermediate	1.25 *(8)*	0.98 (6)	0.81 (4)	1.67 (2)	8.33 (2)	
- University	90.44 *(577)*	91.04 (559)	93.52 (462)	80.83 (97)	75.00 (18)	
- Postgraduate	1.57 *(10)*	1.63 (10)	1.21 (6)	3.33 (4)	0	
**Employment Status**						<0.0001
- Student	31.35 *(200)*	181 (29.48)	24.90 (123)	48.33 (58)	79.17 (19)	
- Working	68.65 *(438)*	433 (70.52)	75.10 (371)	51.67 (62)	20.83 (5)	

* Indicates continuous variables represented as mean (SD). Categorical variable values in each cell are represented as percentage (sample size). ** Significance of differences evaluated using χ_2_ test for categorical variable and t-test or ANOVA for continuous variable. Significance accessed if p-value < 0.05 in the above table. All were significant at 0.05 except smoking status, which had p-value 0.157 for diabetic vs. non-diabetic class and p-value 0.311 for diabetic classification (non, pre and diabetic).

Moreover, the BMI (according to WHO criteria) [34] ranged from 15–50 kg/m^2^, with average BMI being 25.7 (SD = 6.25); whereas, waist circumferences ranged from 20cm to 125cm. About 54.1% of females had a normal BMI, 23.4% were overweight, and 22.6% were obese, while 25 women had BMI higher than 40 kg/m^2^. The mean BMI was significantly higher among diabetics (32.09, SD = 7.41) compared to prediabetics (28.14, SD = 6.42) and nondiabetics (24.77, SD = 5.79). Similarly, the prediabetics also had a higher mean BMI compared to nondiabetics (p-value < 0.0001).

Furthermore, we found that 113 females (17.71%) had high WC (greather than 88cm) and 3 females (0.5%) had high WC but normal BMI. A significant positive correlation (r = 0.79) was found between BMI and WC. The mean waist circumference was significantly higher among diabetics (93.23, SD = 18.06) compared to prediabetics (82.18, SD = 13.57) and nondiabetics (73.55, SD = 12.40). Similarly, the waist circumference of prediabetics was higher compared to nondiabetics (p-value < 0.0001).

Out of 638 females, 500 (78.37%) were single and 138 (21.63%) were married. There were 11.6% and 29% married females who had diabetes and prediabetes, respectively; whereas 1.6% and 16% of unmarried females had diabetes and prediabetes, respectively. Hence, married females were more likely to have diabetes and prediabetes compared to single females (p-value < 0.0001). A majority of the females in the study were nonsmokers. Only six females reported being current or ex-smokers, and only one among them was diabetic, with no smokers in the prediabetes group. Therefore, smoking status did not have any significant relationship with diabetes (p-value = 0.311).

Aaround 92% of the participants had an educational level at university or higher, and only 8% had an intermediate or less education level. Out of 638 participants, 75% of the diabetic cases were university or postgraduate educated; whereas, 84.2% of the prediabetic cases had university level or postgraduate level education. Participants who had a primary education and diabetes or prediabetes was 27.3% and 54.6%, respectively; whereas, 3.1% and 17.2% with university or postgraduate education had diabetes and pre-diabetes, as shown in Table 2. The prevalence of diabetes and pre-diabetes is shown to be decreasing proportionally with education attainment level, except for secondary education (p < 0.0001).

**Table 2:** Crude prevalence and trait-specific prevalence of diabetes and prediabetes in the female Al Kharj population.

	Prediabetes (n = 120)	95% CI	Diabetes (n = 24)	95% CI

**Overall**	18.81	*15.77–21.85*	3.76	*2.28–5.24*
**Age (years)**				
- 18–24	14.54	*11.47–17.61*	1.38	*0.36–2.38*
- 25–44	34.48	*25.78–43.19*	10.34	*4.77–15.92*
- 45–60	46.15	*17.89–74.41*	38.46	*10.88–66.04*
**BMI**				
- Normal	12.75	*9.22–16.29*	0.87	*0.1–1.85*
- Overweight	20.81	*14.25–27.36*	2.68	*0.08–5.29*
- Obese	31.25	*23.64–38.86*	11.81	*6.5–17.10*
**Waist Circumference**				
- ≤88 cm	16.19	*13.03–19.35*	2.10	*0.87–3.32*
- >88 cm	30.97	*22.39–39.55*	11.50	*5.58–17.42*
**Smoking Status**				
- Never Smoker	18.99	*15.92–22.05*	3.64	*2.17–5.10*
- Ex-Smoker	–	–	–	
- Current Smoker	–	–	20	*0.01–59.27*
**Marital Status**				
- Married	28.99	*21.37–36.60*	11.59	*6.22–16.97*
- Single	16.00	*12.78–19.22*	1.60	*4.97–2.70*
**Education Level**				
- Primary	54.55	*23.63–85.47*	27.27	*0.01–54.93*
- Secondary	34.38	*17.62–51.13*	3.13	*0.01–9.26*
- Intermediate	25	*0.01–57.14*	25	*0.01–57.14*
- University or Postgraduate	17.2	*14.14–20.27*	3.07	*1.67–4.47*
**Employment Status**				
- Student	29	*22.68–35.31*	9.50	*5.42–13.58*
- Working	14.16	*10.88–17.42*	1.14	*0.14–2.14*

Table 2 shows the crude and trait-specific prevalence for diabetes and prediabetes among females at Al Kharj, which was found to be 3.8% and 18.8%, respectively. Additionally, age-specific prevalence and other trait-specific prevalence with 95% confidence interval are also presented. The prevalence of diabetes is shown to be increasing proportionally with increasing age: 1.4%, 10.3%, and 38.5% for age group 18–24, 25–4, and 45–60, respectively. Similarly, prevalence of prediabetes is shown to be increasing proportionally with increasing age: 14.5%, 34.5%, and 46.2% for age group 18–24, 25–44, and 45–60, respectively. Prevalence of diabetes is shown to be increasing proportionally with increasing levels of BMI: 0.9%, 2.7%, and 11.8% for normal, overweight, and obesity levels, respectively. Prevalence of prediabetes is shown to be increasing proportionally with increasing levels of BMI: 12.75%, 20.8%, and 31.3% for normal, overweight and obesity levels, respectively. Among females with high waist circumference, the prevalence of prediabetes was higher at 31% and diabetes at 11.5%. Diabetes and prediabetes were found to be more prevalent in older age, higher BMI, and high waist circumference groups. We also found a prevalence ratio of 2.9, 5.4, and 1.9 for having diabetes compared to prediabetes given an individual with high WC (vs. WC < 88 cm), being obese (vs. Normal BMI), and being overweight (vs. Normal BMI), respectively.

With respect to employment status, some were students (31.4%) and others were working (68.7%). Between those, 79.2% of individual who had diabetes were students; whereas, only 20.1% of the diabetics were working. On the contrary, 48.3% of prediabetic individuals were students; whereas, 51.7% prediabetics were working. Table 2 illustrates the 29% and 14.2% prevalence of prediabetes for students and workers. We found 9.5% and 1.1% prevalence of diabetes for students and workers. This indicates that diabetic and prediabetic students are more likely to have diabetes compared to workers (p-value < 0.0001). We also found the prevalence ratio of 4.1 for diabetics compared to prediabetics given an individual being either a student or worker.

Table 3 demonstrates the univariate and multivariable analysis reporting unadjusted and adjusted odds ratio using log-binomial regression for diabetic and nondiabetic results, together with using multinomial logistic regression modeling for prediabetic and diabetic levels.

**Table 3:** Regression modeling for diabetic classification and its risk factors in te female Al Kharj population.

	Unadjusted OR Diabetes (vs. Total Nondiabetes)	Adjusted OR* Diabetes (vs. Total Nondiabetes)	By diabetic classification			

			Unadjusted OR		Adjusted OR*	

			Prediabetes (vs. Nondiabetes)	Diabetes (vs. Nondiabetes)	Prediabetes (vs. Nondiabetes)	Diabetes (vs. Nondiabetes)

**Age (years)**						
- 18–24	1	–	1	1	–	–
- 25–44	**7.52** *(3.03–18.69)*	–	**3.62** *(2.27–5.76)*	**11.46** *(4.35–30.20)*	–	–
- 45–60	**27.97** *(10.21–76.55)*	–	**17.35** *(3.43–87.61)*	**152.86** *(25.22–926.47)*	–	–
**BMI**						
- Normal	1	1	1	1	1	1
- Oveweight	3.09 *(0.70–13.62)*	1.84 *(0.37–9.08)*	**1.84** *(1.11–3.06)*	3.49 *(0.77–15.82)*	1.36 *(0.80–2.33)*	1.59 *(0.32–7.91)*
- Obese	**13.58** *(4.04–45.61)*	**5.47** *(1.54–19.36)*	**3.72** *(2.30–6.02)*	**20.59** *(5.89–71.98)*	**2.35** *(1.38–3.99)*	**6.67** *(1.68–26.42)*
**Waist Circumference**						
- ≤88cm	1	1	1	1	1	1
- >88cm	**5.49** *(2.53–11.94)*	1.65 *(0.58–4.75)*	**2.72** *(1.69–4.36)*	**7.80** *(3.35–18.14)*	1.53 *(0.89–2.63)*	1.98 *(0.68–5.73)*
**Smoking Status**						
- Never Smoker	1	1	–	1	–	1
- Current Smoker or Ex-Smoker	4.58 *(0.73–28.65)*	**4.62** *(1.81–11.80)*	–	4.26 *(0.48–37.91)*	–	4.16 *(0.28–61.50)*
**Marital status**						
- Single	1	1	1	1	1	1
- Married	**7.25** *(3.17–16.58)*	2.34 *(0.73–7.55)*	**2.51** *(1.61–3.93)*	**10.05** *(4.16–24.25)*	1.09 *(0.61–1.93)*	2.11 *(0.70–6.34)*
**Education Level**
- Primary	1	1	1	1	1	1
- Secondary	**0.11** *(0.01–0.99)*	1.23 *(0.13–11.53)*	0.18 *(0.03–1.07)*	**0.03** *(0.01–0.49)*	1.07 *(0.16–7.32)*	0.81 *(0.04–15.15)*
- Intermediate	0.92 *(0.20–4.28)*	3.49 *(0.70–17.42)*	0.17 *(0.02–1.72)*	0.33 *(0.03–3.92)*	0.34 *(0.03–3.72)*	1.62 *(0.11–22.98)*
- University or Postgraduate	**0.11** *(0.04–0.33)*	**3.74** *(1.03–13.64)*	**0.07** *(0.01–0.36)*	**0.02** *(0.01–0.16)*	0.82 *(0.13–5.27)*	2.78 *(0.28–27.83)*
**Employment status**						
- Student	1	1	1	1	1	1
- Working	**0.12** *(0.05–0.32)*	0.35 *(0.10–1.20)*	**0.35** *(0.23–0.53)*	**0.09** *(0.03–0.24)*	0.71 *(0.42–1.20)*	0.39 *(0.11–1.33)*

* Adjusted by continuous age variable. Bold numbers indicate statistical significance (p < 0.05).

On univariate analysis, the following variables had a significant association with prediabetes and diabetes: age, overweight, obesity, waist circumference, marital status, employment, and education. Similarly, for diabetes compared to nondiabetes in the multinomial logistic regression model, age, obesity, waist circumference, employment status, education, and marital status were significantly associated with diabetes. However, on multivariable analysis, after adjusting for age, only obesity had a significant association with both prediabetes and diabetes compared to nondiabetes. We observed the prediabetics were 2.35 times more likely to be obese compared to nondiabetics (95% CI, SD = 1.38–3.99). Similarly, students compared to the working population are at more risk for diabetes and prediabetes. Similarly, diabetics were 6.67 times more likely to be obese compared to nondiabetics (95% CI, SD = 1.68–26.42).

## 4. Discussion

Diabetes mellitus is an important public health issue worldwide. It is a chronic disorder having vast environmental and genetic effects. It continues to be a worldwide health crisis in the 21st century, and it is estimated that about 300 million individuals will be affected with diabetesby 2025 [37]. If not diagnosed and treated at early stages, it may give rise to overwhelming complications. The current study was conducted to create insight into the prevalence and determinants of prediabetes and diabetes among females of Al-Kharj. In our study, the prevalence of diabetes and prediabetes among females from Al Kharj was 3.8% and 18.8%, respectively. The overall increase in prevalence of diabetes in Saudi Arabia from 16.5% (2010) to 18.8% (2030) is alarming [38]. In our study, diabetes and prediabetes status was well-defined using HbA1c cutoff levels of less than 5.7% (nondiabetics), greater than 5.7% but less than 6.5% (prediabetes), and greater than or equal to 6.5% (diabetes mellitus) as per the American Diabetes Association 2016 [36]. However, previous studies used fasting plasma glucose alone for the diagnosis of diabetes [15,39], which may genuinely underestimate the actual magnitude of the problem in Saudi Arabia. Additionally, few studies include only the Saudi nationality, hence they may not provide a complete picture of the prevalence of DM [1,17,20,22,40,41]. An international expert committee suggested the use of HbA1c, glycated hemoglobin A1C, as a diagnostic tool for diabetes [15,17]. This shows the average blood level of glucose for the past 3 months and requires treatment if it is 6.5% or higher [6].

The mean HbA1c in our study participants was 5.44, with a range from 2.3 to 12.7. However, the mean HbA1c levels among diabetics was 7.72 (2.12), among prediabetics was 5.87 (0.17), and among nondiabetics was 5.23 (0.32). A study conducted in Japan revealed HbA1c levels ranging from 5.7% to 6.4% [42]. The HaA1c range was much higher among our population, signifying a need for interventions to reduce the prevalence of diabetes.

The literature shows that lifestyle modification is the cornerstone for managing diabetes [15,17]. The first step includes increasing physical activity and improving diet, consequntly controlling obesity. These are the two most significant modifiable factors [43]. The beneficial effects of lifestyle interventions have been confirmed among the Chinese [44], Swedish [45], Finish [46], American [43], Asian [47,48], and Saudi populations [17]. Pharmacological interventions include antidiabetes drugs, including Biguanides (Metformin), Thiazolidinediones, α-glucosidase inhibitors. The GLP-1 analogues and insulin secretagouges are also prescribed according to their indications [15]. This indicates the need for more focused awareness campaigns across the nation. An integrated care intervention was provided to the patients in primary care in Riyadh, Saudi Arabia. It consisted of an intensified and patient-specific multidisciplinary care program by a team that included a family physician, a nurse, a clinical pharmacist, a dietician, a diabetic educator, and a social worker, which resulted in a 20% improvement in glycaemic control as measured by reduction in HbA1c and fasting blood glucose [49]. Another strategy was the use of a mobile diabetes management system [50].

In our study, obesity was significantly associated with diabetes and prediabetes. Obesity is the root cause for a majority of noncommunicable diseases (NCDs) in the world and is becoming a public health issue. It is increasing at an alarming rate, as reported by studies conducted in China [51] and Saudi Arabia [23,24,28,40]. In Saudi Arabia, 33.8% of women in Jeddah were determined to be obese in Jeddah: 25.1% had a WC greater than 88 cm, 47.1% had a WC greater than 80 cm, and 31.2% were physically inactive [52]. Abdominal obesity, specifically, is the second most significant predictor of both diabetes and prediabetes [1]. Similarly, in our study 23.35% of the participants were found to be overweight, while 22.57% were obese, and 17.71% had high WC (i.e., >88 cm), predisposing them to various NCDs, including DM. In Jeddah, increased WC was found strongly associated with prediabetes, specifically in females with DM [1]. As shown in our study, diabetic female participants had a higher mean WC and BMI. Obesity is a modifiable risk factor for prevention and management of diabetes. Therefore, it is imperative that intervention programs be developed and receive major consideration for DM prevention [53].

Type of job may affect the occurrence of diabetes due to effects of behavior or metabolic regulation and because of the level of earning and physical activity. In our study, students were more likely to have diabetes compared to working females. Two of the studies revealed a high inactivity level was present among Saudi women, pertaining to the international recommendation for minimum activity [54]. Another study observed female nurses working fewer than 20 hours per week had a lesser risk of diabetes, while those working overtime (41 or more hours per week) had an elevated risk of diabetes compared with women working 21 to 40 hours per week in paid employment [55]. A study in the Delhi population demonstrated the higher-income group had poor glycemic control [56]. However, other studies found less prevalence of DM with increasing occupational activity for both sexes [57].

Around 44% of the females in our study were university educated. However, contrasting findings were reported by a study conducted in Europe, which demonstrated individuals with a lower education background had a higher rate of DM compared to those with a higher level of education [58].

Moreover, smoking is an independent risk factor. Nevertheless, due to cultural and religious boundaries, fewer females in Saudi Arabia smoke or, if they smoke, they feel hesitant to reveal their smoking status. There were not enough smokers to detect smoking effects in our study population. Only six participants in our study reported being current or ex-smokers; therefore, no clear indication for smoking association was found with diabetes status; however, more smokers were found in the nondiabetics. A study found no relationship between diabetes and smoking status of patients [59]. Another study revealed they found minimal information on the effectiveness of smoking cessation interventions [60]. However, contradictory findings were observed from a study conducted in Ireland, which described cigarette smoking was found to be significantly associated significantly with an enhanced risk of diabetes, even though other potential confounders like BMI and age were adjusted during analysis [61]. Conversely, another study demonstrated adverse effects of smoking on diabetes and its complications, together with rendering it as a target for diabetes control [62].

In our study, we found married females to be more likely to be diabetic. Similar findings were reported by a study conducted in Florida, where marital status and education level were found to be significant predictors, along with other factors, for DM and prediabetic status [7]. They concluded a higher proportion of married individuals, compared to unmarried prediabetic or diabetic. Conversely, studies from Iran [63] and Bangladesh [64] could not find any significant relationship between marital status and diabetes. To address these findings, it could be suggested that married individuals have a high degree of responsibilities, due to which they usually have no time for physical activity. This sedentary lifestyle ultimately leads to obesity and a risk of diabetes. In a similar context, marital status was significantly associated with obesity in Greek adults [65] in contrast to Malaysians [66] and Americans [67], who report increased physical activity after marriage. Various risk factors have been identified by different regions of the world for DM and prediabetes. Hence, modifiable risk can be controlled to some extent, which may contribute to the development, as well as advancement, of the disease.

### 4.1. Limitations and Strengths

There were several strengths of our study. Our robust sampling technique (i.e., multistage stratified cluster sampling) was used. Hence, we were able to recruit working and student female participants belonging to different demographic backgrounds. All institutes from Al-Kharj were included in the sampling frame. Moreover, a robust tool for assessing diabetes status (i.e., HbA1c) was used, which has been used as an authentic marker for evaluating the presence of this disease. Because fasting plasma, glucose can usually detect only a certain proportion of individuals with DM and prediabetes, HbA1c is a much more robust tool for assessing diabetes status.

However, our study had a few limitations. First, our study was conducted in Al-Kharj and not in other regions of Saudi Arabia; therefore, generalizing our results to Saudi Arabia might be questionable. However, the study can be generalized to the studying and working force of Al-Kharj. We use the WHO recommended cut-offs for BMI and WC, which might be different for the Saudi population due to geographical differences and dietary habits. Hence, it is imperative to establish BMI cut-offs specifically for the population in our region. Additionally, family history, concomitant illnesses, habitual dietary intake, and ethnicity should have been considered while evaluating the predictors of DM and prediabetes.

## 5. Conclusion

Our study results indicate the prevalence of diabetes and prediabetes among females from Al Kharj (3.8% and 18.8%, respectively). The diabetic and prediabetic female participants, compared to nondiabetics, had higher mean BMI and WC, were older in age, employment status, and education level, and were married. In the context of the findings of our study and keeping in view the burden of this disease globally and in our population, it has now become extremely important to understand these factors and encourage health-promoting behaviors to construct effective interventions.

## 6. Implications and Recommendations

With DM approaching an epidemic in Saudi Arabia, it is crucial to promote preventive programs to decrease its burden. Practitioners and health policy professionals should devote attention towards promotion of public awareness, regular screening, early diagnosis, and prompt intervention.

A considerable number individuals with diabetes or prediabetes remain undiagnosed, and that rate varies across countries due to varying health systems and surveillance systems. Without proper evaluation, this can lead to major health issues and complications in the future. Hence, extensive screening for diabetes is recommended at the primary level of health care, where general practitioners can play a vital role by evaluating the high-risk groups based on general characteristics, family history, risk factors, and by using effective screening tools for early detection. Health education and counselling sessions should be conducted during routine check-ups in primary health care clinics, universities, work places, and medical conferences. Healthcare workers, general practitioners, and the social media can play a vital role in disseminating information. It is also imperative to establish national cut-offs for measures of indicators for obesity. Moreover, future cohort studies should be conducted to thoroughly investigate anthropometric measurements and other risk factors to improve the health of Saudi females for future generations.

## Data Accessibility Statement

The data used to support the findings of this study were provided by Prince Sattam Bin Abdulaziz University under license and cannot be made freely available. Access to these data will be considered by the author upon request, with permission of Prince Sattam Bin Abdulaziz University.
